# Epitope Density Influences CD8^+^ Memory T Cell Differentiation

**DOI:** 10.1371/journal.pone.0013740

**Published:** 2010-10-29

**Authors:** Julie Leignadier, Nathalie Labrecque

**Affiliations:** 1 Maisonneuve-Rosemont Hospital Research Center, University of Montreal, Montreal, Quebec, Canada; 2 Department of Microbiology and Immunology, University of Montreal, Montreal, Quebec, Canada; 3 Department of Medicine, University of Montreal, Montreal, Quebec, Canada; New York University, United States of America

## Abstract

**Background:**

The generation of long-lived memory T cells is critical for successful vaccination but the factors controlling their differentiation are still poorly defined. We tested the hypothesis that the strength of T cell receptor (TCR) signaling contributed to memory CD8^+^ T cell generation.

**Methodology/Principal Findings:**

We manipulated the density of antigenic epitope presented by dendritic cells to mouse naïve CD8^+^ T cells, without varying TCR affinity. Our results show that a two-fold decrease in antigen dose selectively affects memory CD8^+^ T cell generation without influencing T cell expansion and acquisition of effector functions. Moreover, we show that low antigen dose alters the duration of the interaction between T cells and dendritic cells and finely tunes the expression level of the transcription factors Eomes and Bcl6. Furthermore, we demonstrate that priming with higher epitope density results in a 2-fold decrease in the expression of Neuron-derived orphan nuclear receptor 1 (Nor-1) and this correlates with a lower level of conversion of Bcl-2 into a pro-apoptotic molecule and an increased number of memory T cells.

**Conclusions:**

Our results show that the amount of antigen encountered by naïve CD8^+^ T cells following immunization with dendritic cells does not influence the generation of functional effector CD8^+^ T cells but rather the number of CD8^+^ memory T cells that persist in the host. Our data support a model where antigenic epitope density sensed by CD8^+^ T cells at priming influences memory generation by modulating Bcl6, Eomes and Nor-1 expression.

## Introduction

During an immune response, antigen (Ag)-specific naïve T cells undergo massive proliferation and differentiate into effectors that eliminate the pathogen. After Ag clearance, 90–95% of effector T cells die while a few differentiate into long-lived memory T (Tm) cells. The efficient protection provided by Tm cells is due to both the increased number of Ag-specific T cells as well as their enhanced sensitivity upon Ag re-exposure [1]. Therefore, Tm cell development is critical for the control of recurrent infections and for the success of vaccination. A better understanding of the molecular events leading to Tm cell generation is crucial to improve vaccination. However, there is limited information regarding the signals that dictate Tm cell generation and we still do not known if the strength of T cell receptor (TCR) signaling and the affinity/avidity of the TCR contribute to the development of CD8^+^ Tm cells.

While strong experimental evidences exist for a role of the strength of TCR engagement for CD4^+^ Tm cell generation [2], [3], [4] such evidences are scarce or against this concept in the development of CD8^+^ Tm cells [5], [6]. A recent study has evaluated the role of TCR affinity for the generation of effector and memory CD8^+^ T cells [6]. Using altered peptide ligands, they have shown that Ags with very low affinity for the TCR are able to induce the complete differentiation of naïve CD8^+^ T cells into effector and memory cells. However, the strength of TCR-ligand interaction affected the level of T cell expansion [6]. To evaluate the contribution of the strength of TCR signaling in the generation of effector and memory CD8^+^ T cells, rather than modifying the nature of Ag, we decided to manipulate the level of Ag presentation by dendritic cells (DCs) to naïve CD8^+^ T cells while maintaining constant the affinity of the TCR for its ligand. Unexpectedly, we observed that lowering the avidity of the TCR-MHC-peptide interactions by decreasing Ag dose by only two-fold strongly affected the generation of CD8^+^ Tm cells without impacting effector generation. Moreover, we showed that lower density of MHC-peptide complexes at the surface of DCs alters the quality of T-DC interaction. Consequently, the induction of the molecular program required for CD8^+^ Tm cell development is altered as shown by a fine tuning in expression level of the transcription factors Eomes and Bcl6. Furthermore, our results showed that higher level of TCR engagement at priming is necessary to promote the survival of memory precursor CD8^+^ T cells during the contraction phase of the response. Indeed, we identified Neuron-derived orphan nuclear receptor 1 (Nor-1) as a possible new pathway controlling Ag-specific CD8^+^ T cell survival during contraction. Altogether, our data supports a model where the avidity of the TCR for its ligand influences CD8^+^ Tm cell generation.

## Results

### Epitope density influences CD8^+^ T cell fate

To study the role of TCR signaling strength for CD8^+^ Tm cell development, we chose to only vary the dose of Ag while keeping TCR affinity and inflammation constant. To eliminate any influence of TCR affinity, we adoptively transferred ovalbumin (OVA)-specific CD8^+^ T cells bearing a monoclonal TCR specific for the K^b^-SIINFEKL complex. To maintain constant and low levels of inflammation, we immunized these mice with DCs while we manipulated Ag dose by loading the DCs with different densities of major histocompatibility (MHC)-peptide complexes. Furthermore, DC immunization favors the early generation of Ag-specific CD8^+^ T cells with a memory precursor phenotype [7], [8], [9]. Although these cells have a memory precursor phenotype at the peak of the response, they also acquire effector functions and go through a normal contraction phase [7], [9]. This suggests that they must undergo further maturation to become long-lived CD8^+^ Tm cells and that only a fraction of the memory precursors has acquired the proper genetic program to become long-lived Tm cells. Therefore, this system allows us to evaluate if the strength of TCR signaling at priming impinges on the optimal differentiation of memory CD8^+^ precursors into long-lived Tm cells.

**Figure 1:**
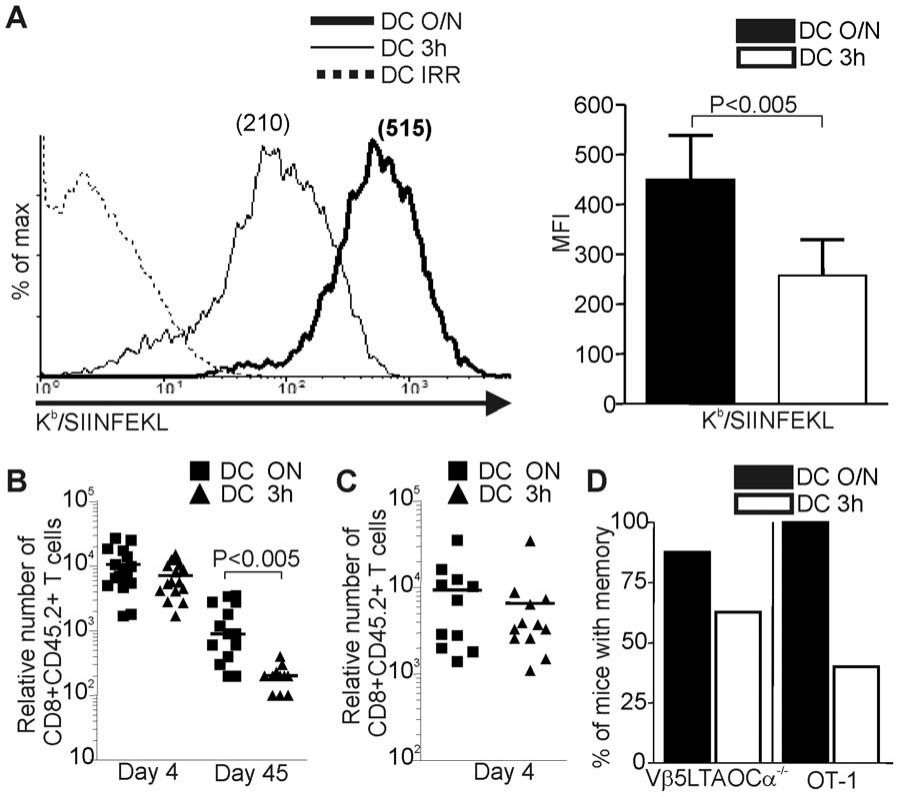
Epitope density influences CD8^+^ Tm generation. A**.** The duration of peptide loading on DCs affects MHC-peptide expression. Representative histogram of K^b^-SIINFEKL expression by DCs loaded 3 h (DC 3 h) or O/N (DC O/N) with SIINFEKL (left); dashed line, DCs pulsed with an irrelevant peptide (DC IRR). The mean fluorescence intensity (MFI) of K^b^-SIINFEKL expression is indicated on the top of the respective histogram. Right panel, compilation of the MFI of K^b^-SIINFEKL expression for DC O/N versus DC 3 h. Results are from 13 independent experiments. B. The number of effector (day 4) and memory (day 45) OVA-specific T cells recovered from LNs after immunization with DC O/N or DC 3 h is shown. Number of Ag-specific T cells was normalized to one million cells to correct for LN size variation. Each dot represents a mouse. Mice in which no Tm cells were generated were not included. C. The number of OVA-specific effectors recovered from the spleen (day 4) after immunization with DC O/N or DC 3 h is shown. Each dot represents a mouse. D. The percentage of mice with Tm cells is shown. Statistical analysis was done using a Student's *t* test.

To generate DCs loaded with different densities of MHC-peptide complexes, bone marrow derived mature DCs were pulsed overnight (DC O/N) or 3 h (DC 3 h) with the SIINFEKL peptide. Very importantly, the DCs were kept in culture and were activated with LPS for the same amount of time to obtain DCs with similar phenotype except for Ag density. DC O/N express two-fold more K^b^-SIINFEKL complexes than DC 3 h (Figure 1A) as measured using the 25.D1.16 mAb that specifically recognizes this peptide-MHC complex [10]. Importantly, the two types of DCs express similar level of MHC class I and II molecules, as well as CD86 and produce similar amount of interleukin (IL)-12 (Figure S1). Thus, the only difference between DC O/N and DC 3 h is the amount of K^b^-SIINFEKL they express. These DCs were used to immunize recipient mice (CD45.1^+^) which have been adoptively transferred with OT-I or Vβ5LTAOCα^−/−^ T cells (CD45.2^+^) expressing a TCR specific for K^b^-SIINFEKL [11], [12]. We always transfer female T cells into female mice followed by immunization with male DCs to provide the T cell help necessary to generate functional CD8^+^ Tm cells [1]. Four days after immunization, a superficial lymph node (LN) was removed by surgery to evaluate the number of Ag-specific CD8^+^ T cells generated. As shown in Figure 1B and Figure S2, the amount of peptide-MHC complexes expressed by DCs did not influence the expansion phase of OVA-specific T cells. Moreover, similar number of effectors was generated in the spleen (Figure 1C) and tertiary sites (Figure S3A). In contrast, the level of Ag presentation to naïve CD8^+^ T cells impacted Tm cell differentiation (Figure 1B and D). OVA-specific CD8^+^ Tm cells were not generated in all mice that were immunized with DC 3 h (6/16) while they were almost always generated in mice immunized with DC O/N (16/19; Figure 1D). Moreover, immunization with DC 3 h generated seven times fewer CD8^+^ Tm cells in the LNs (Figure 1B and Figure S2) and four times less in tertiary sites (Figure S3B). The number of CD8^+^ Tm cells generated was also reduced in the spleen (Figure S3C). Furthermore, with both Ag doses, Tm cells had a central memory phenotype (Figure S4A). In addition, immunization with DCs loaded with an irrelevant peptide did not induce OVA-specific CD8^+^ T cell response (Figure S2). These results show that a stronger TCR signal is necessary for the efficient development of CD8^+^ Tm cells but not for the expansion of Ag-specific CD8^+^ T cells.

### TCR engagement level does not alter CD8^+^ T cell response kinetic

**Figure 2:**
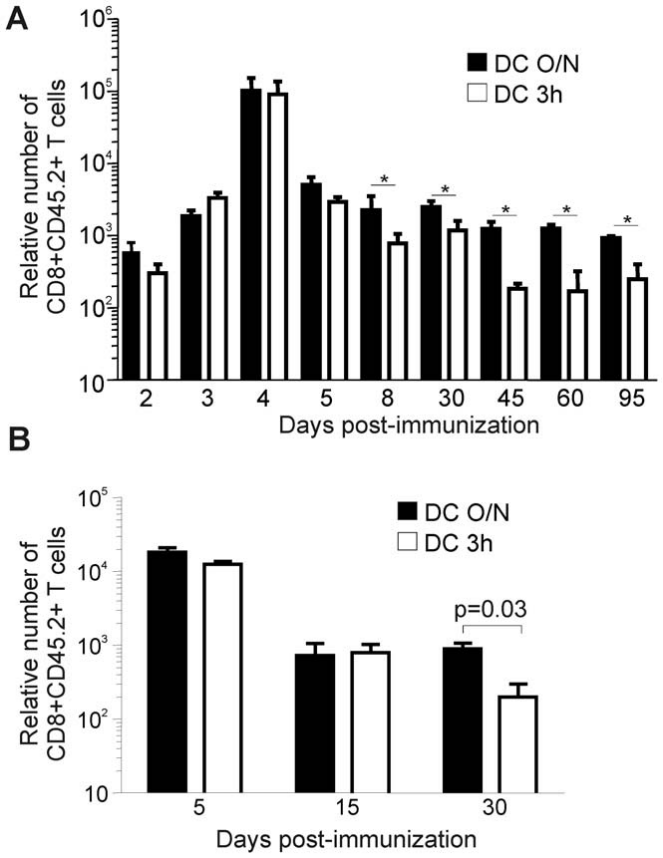
Epitope density does not prolong CD8^+^ T cell expansion. A. The number of CD8^+^CD45.2^+^ T cells was quantified over time after immunization with DC O/N and DC 3 h. LNs were sequentially removed to follow T cell response. The number of CD8^+^CD45.2^+^ T cells was normalized to one million cells to correct for LN size variation (relative number). * p<0.05. Results are pooled from 2 independent experiments with 3 mice per group. B. Generation of CD8^+^ Tm cells is also affected by epitope density when naïve T cell precursor frequency is low. 10^4^ OT-I T cells were adoptively transferred. The relative number of Ag-specific T cells recovered over time from the LNs is shown. Results from one representative experiment out of two are shown with 3 mice per group. Statistical analysis was done using a Student's *t* test.

The size of the Tm cell pool generated after Ag encounter is usually directly proportional to the extent of T cell expansion [13]. Thus, it is possible that immunization with high Ag dose prolongs T cell expansion which will generate more Ag-specific T cells leading to the generation of more CD8^+^ Tm cells. However, we observed a similar kinetic of expansion where the peak of the response is at day 4 for both types of immunization (Figure 2A). Moreover, the contraction phase is initiated at the same time in the two OVA-specific populations but is more pronounced with DC 3 h (Figure 2A). Very importantly, similar results were obtained with a low frequency (10^4^) of naïve T cell precursors (Figure 2B and Figure S5) indicating that the use of a high frequency of naïve precursors did not limit the expansion of T cells after immunization with DCs presenting a higher density of epitope. These results suggest that epitope density on DCs or TCR-ligand avidity does not regulate Ag specific CD8^+^ T cell proliferation after priming but influences their survival during contraction.

### Phenotype and functions are not influenced by the amount of Ag encountered by naïve CD8^+^ T cells

The inefficient CD8^+^ Tm cell development after immunization with DCs presenting low Ag density could result from an altered phenotype of Ag-specific T cells at the peak of the response. However, we did not observed any differences in the expression of cell surface (Ly6C, Sca-1 and 4-1BB), activation (CD44, 1B11 and CD5) and migratory (CD62L and CCR7) markers, as well as cytokine receptors (CD25, CD122 and CD127) (Figure S4). In both instances, the effectors generated did not acquired KLRG-1 expression (not shown) and maintained high level of CD127 (Figure S4). This suggests that the reduced production of CD8^+^ Tm cells after immunization with DC 3 h is not due to the acquisition of a short-lived effector phenotype by Ag-specific CD8^+^ T cells at the peak of the response. Furthermore, effector functions were not affected by epitope density (Figure S6). These results suggest that the 10-fold reduction in CD8^+^ Tm cell generation with low level of Ag presentation is not due to the improper differentiation of naïve CD8^+^ T cells into effectors.

### Epitope density dictates the quality of T-DC interaction

**Figure 3:**
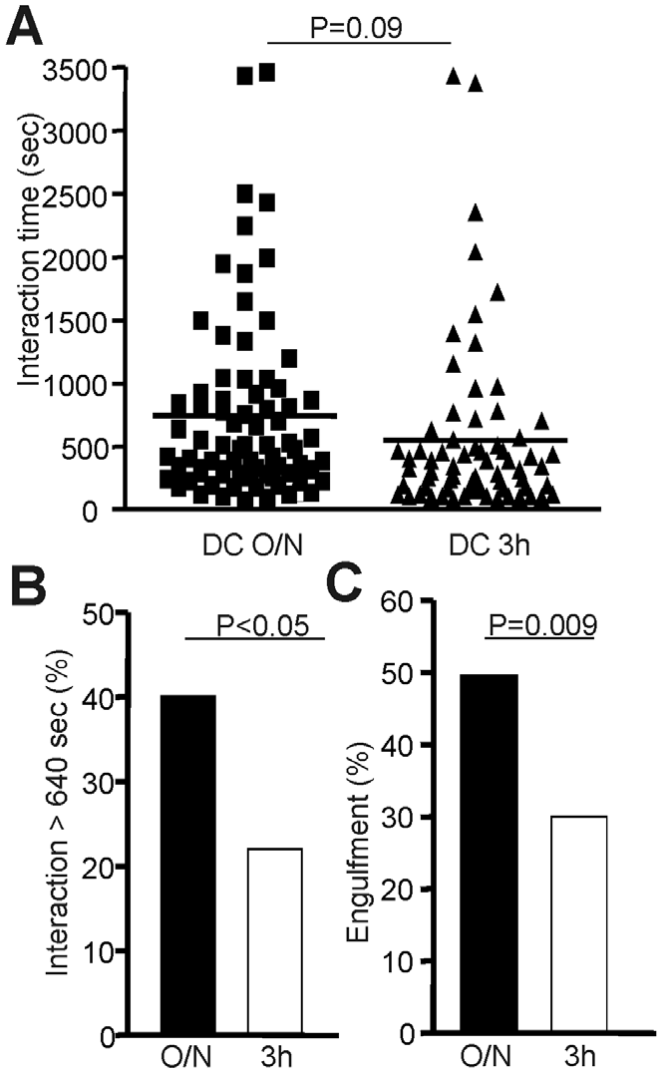
Epitope density dictates the duration and the quality of T-DC interaction. A. The duration of contact between naïve CD8^+^ T cells and DC O/N or DC 3 h was measured *in vitro* using time-lapse microscopy. B. The percentage of naïve CD8^+^ T cells interacting for more then 640 seconds with DCs is shown for DC 3 h or DC O/N. C. The proportion of T-DC interaction that leads to engulfment of naïve CD8^+^ T cells by DCs is shown for DC O/N or DC 3 h. Statistical analysis was done using a Mann-Whitney test.

The variation in the level of Ag presented to naïve T cells could influence the time of interaction between naïve T cells and DCs, a key parameter for the induction of T cell response [14], [15], [16]. Furthermore, it was shown that a reduction in the interaction time between DCs and naïve CD8^+^ T cells due to ICAM-1 deficiency resulted in poor CD8^+^ Tm cell generation [16]. Therefore, we have measured by time-lapse microscopy the duration of contact between naïve CD8^+^ T cells and DC O/N or DC 3 h. The interaction time between naïve OVA-specific CD8^+^ T cells and DCs tends to be lower with DC 3 h (Figure 3A). Importantly, the percentage of naïve CD8^+^ T cells interacting for more than 640 seconds is higher with DCs loaded O/N with the antigenic peptide (Figure 3B). Long-lasting T-DC interaction leads to an intimate contact (engulfment) between T cells and DCs and is a key event controlling T cell activation [14], we evaluated the impact of the Ag dose on the frequency of T-DC interaction leading to engulfment. The frequency of T cell engulfment by DCs was proportional to the level of Ag presented by DCs to naïve CD8^+^ T cells (50% for DC O/N versus 30% for DC 3 h; Figure 3C). Thus, we conclude that a higher density of Ag presented by DCs promotes a long-lasting T-DC interaction at priming which results in efficient generation of long-lived CD8^+^ Tm cells. Therefore, we propose that engulfment of T cells by DCs is essential to generate an optimal TCR signal that will promote CD8^+^ Tm cell generation.

### The amount of Ag encountered by naïve T cells activates a specific genetic program controlling CD8^+^ Tm cell generation

**Figure 4:**
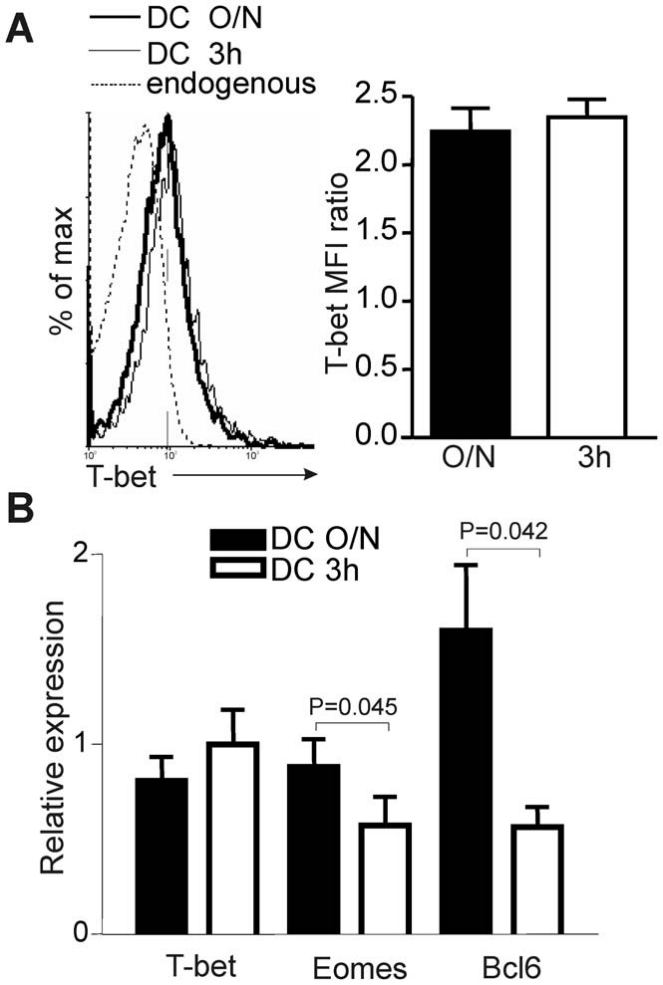
The amount of Ag encountered by naïve CD8^+^ T cells influences transcription factor expression. A. No effect of Ag dose on T-bet expression. The left panel shows intracellular staining for T-bet in effectors at day 4 post-immunization with DC O/N or DC 3 h. The recipient endogenous population was used as a control. The right panel shows compilation of T-bet MFI. B. TCR engagement level finely tunes expression of key transcription factors. At d4 post-immunization, OVA-specific T cells (CD8^+^CD45.2^+^) were sorted from spleen and LNs to perform qPCR analysis. The relative expression of mRNA in effector CD8^+^ T cells obtained after immunization with DC O/N or DC 3 h is shown (normalized to HPRT). 3 independent experiments with 3 mice per group. Statistical analysis was done using a Mann-Whitney test.

Our model puts us in a unique position to decipher the genetic program required for CD8^+^ Tm cell development since manipulating epitope density specifically affects CD8^+^ Tm cell generation. Furthermore, our model also offers the advantage that inflammation level remains constant even when we increase Ag dose. Therefore, we evaluated the expression level of key transcription factors involved in CD8^+^ T cell response. The transcription factor T-bet is necessary for the generation of CD8^+^ Tm cells [17] and its expression level dictate the fate of effector CD8^+^ T cells [8]. Indeed, high T-bet level is associated with the generation of short-lived effectors while low level of T-bet is present in CD8^+^ memory precursors [8]. Therefore, it was possible that a certain level of TCR engagement was necessary to up-regulate T-bet expression. However, at the peak of the response (day 4), T-bet expression was similar in sorted OVA-specific CD8^+^ T cells generated with low and high Ag doses (Figure 4A and B).

In contrast, high level of Eomes expression correlates with CD8^+^ Tm cell development but direct evidence for a role of Eomes is still lacking [18]. At the peak of the response, we observed a two-fold increase in Eomes expression by effectors generated following immunization with DC O/N when compared to DC 3 h (Figure 4B). This suggests that the strength of TCR signaling directly regulate Eomes expression.

The transcriptional repressor Bcl6 regulates B and T cell memory generation [19], [20], [21], [22] but unlike T-bet and Eomes, Bcl6 is not necessary for effector generation [21], [22]. Accordingly, we observed a 1.3–1.6-fold increase in Bcl6 expression in primed Ag-specific CD8^+^ T cells that efficiently become Tm cells when compared to those that do not (Figure 4B). This suggests that a threshold of Bcl6 expression is required for efficient generation of CD8^+^ Tm cells and that the level of TCR engagement controls Bcl6 expression level.

Therefore, we conclude that epitope density at priming influences the genetic signature of Ag-specific T cells which then impinges on their further differentiation into CD8^+^ Tm cells.

### Similar expression of pro- and anti-apoptotic molecules in CD8+ effectors generated with low and high density of Ag

One possible explanation for inefficient Tm cell generation after immunization with DCs presenting Ag at low density could be an increase death rate of Ag-specific effectors. Apoptosis can be induced in effector CD8^+^ T cells by different molecules such as Spi2A, Fas ligand (FasL) and Bim while survival can be promoted by the anti-apoptotic molecules Bcl-2 and Bcl-x_L_. Bcl-2 expression in OVA-specific T cells was not dependent on the amount of TCR engaged with MHC-peptide complexes (Figure S7). Moreover, there was no significant difference in the relative expression of Spi2A, FasL, Bim and Bcl-X_L_ in OVA-specific T cells generated after immunization with DC 3 h or DC O/N (Figure S7). These results show that the severe reduction in CD8^+^ Tm cell generation in our model is not a consequence of inappropriate expression of these pro- and anti-apoptotic molecules.

### Nor-1/Bcl2, a novel pathway controlling effector CD8^+^ T cell fate

**Figure 5:**
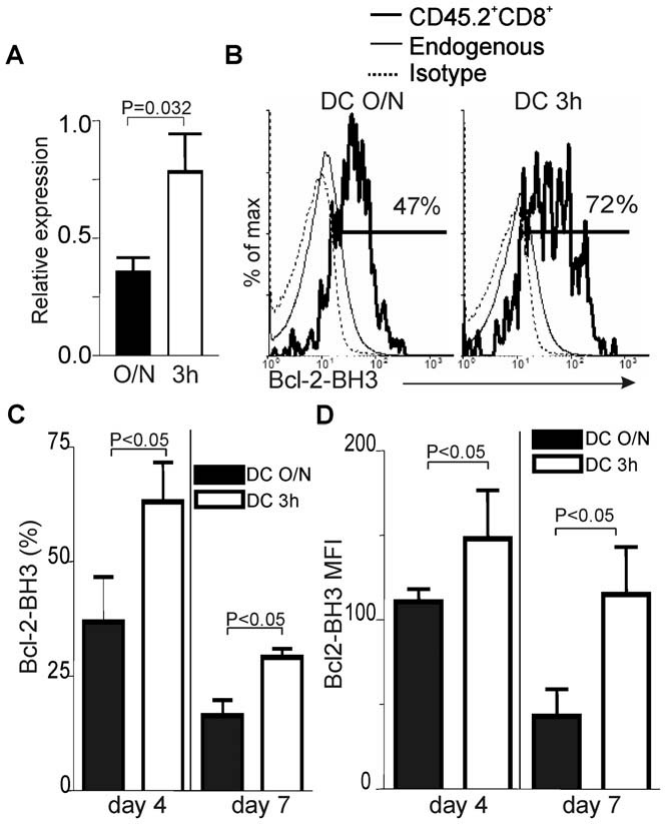
Selective downregulation of Nor-1 expression in effector CD8^+^ T cells generated with high Ag dose. A. Expression of Nor-1 in effector CD8^+^ T cells. Effectors (CD8^+^CD45.2^+^) were sorted from spleen and LNs at d4 post-immunization to perform qPCR. The relative expression of Nor-1 after immunization with DC O/N or DC 3 h is shown (normalized to HPRT). 3 independent experiments with 3 mice per group. B. Epitope density influences the exposure of the BH3 domain of Bcl-2 in CD8^+^ effector T cells. The overlays show staining gated on OVA-specific effector (CD8^+^CD45.2^+^) T cells obtained at day 4 post-immunization with DC O/N or DC 3 h. The controls were isotype and staining of the endogenous population (CD8^+^CD45.2^−^). C and D. The histogram represents the percentage (C) and the MFI (D) of Bcl-2-BH3 expression by Ag-specific T cells at d4 or d7 post-immunization with DC O/N or DC 3 h. 3 independent experiments with 2–3 mice per group. Statistical analysis was done using a Mann-Whitney test for panel A and a Student's *t* test for panels C and D.

Recently, Bevan's group reported that low avidity effector CD4^+^ T cells unable to differentiate into CD4^+^ Tm cells have up-regulated Bim and Nor-1 expression [4]. Nor-1 is a member of the nuclear receptor family of intracellular transcription factor encoded by the *Nr4a3* gene [23]. In the thymus, Nor-1 is associated with apoptosis and negative selection [24]. Furthermore, Nor-1 translocates to the mitochondria after TCR-mediated stimulation in thymocytes [25] where its binding to Bcl-2 exposes the Bcl-2 BH3 domain. This converts Bcl-2 into a pro-apoptotic molecule (thereafter named Bcl-2-BH3) [25]. Then, Bcl-2-BH3 contributes to cytochrome c release leading to apoptosis. The possible role of Nor-1 in T cell apoptosis led us to evaluate its expression level in primed Ag-specific CD8^+^ T cells that are able or not to generate Tm cells. The relative expression of the *Nr4A3* gene was two-fold less in OVA-specific CD8^+^ T cells generated after immunization with DC O/N compared to DC 3 h (Figure 5A). Since Nor-1 activates apoptosis via binding to Bcl-2 and exposition of the BH3 domain of Bcl-2, we evaluated if the higher level of Nor-1 expression promoted the conversion of Bcl-2 into a pro-apoptotic molecule. Using a mAb that recognizes specifically the Bcl-2-BH3 pro-apoptotic form of Bcl-2 [25], we showed that the expression of Bcl-2-BH3 was increased in effectors obtained after immunization with DCs presenting low versus high level of Ag at d4 and d7 post-immunization (Figure 5B–D).

These results show that the level of TCR engagement regulates the expression of Nor-1 which can, via its association with Bcl-2, promote Ag-specific CD8^+^ T cell death. Furthermore, this is the first suggestion that Nor-1 regulates effector CD8^+^ T cell fate.

## Discussion

Our results show that the amount of Ag encountered by naïve CD8^+^ T cells following DC immunization does not influence the generation of functional effector CD8^+^ T cells but rather affects CD8^+^ Tm cell differentiation. In the past, few groups have studied the impact of Ag dose on CD8^+^ Tm cell development. These studies evaluated CD8^+^ T cell response to infection and reported that variation in Ag level selectively affects the number of effectors generated without influencing memory differentiation [5], [26]. The fact that we did not see any effect of Ag dose on the expansion of Ag-specific CD8^+^ T cells could be because we varied by only two-fold the amount of MHC-peptide complexes presented to naïve CD8^+^ T cells. Additionally, the level of inflammation may also account for this difference, where DC immunization induces very low level of inflammation relative to the inflammation induced upon infection. Furthermore, in our experiment, we studied the response of monoclonal, rather than polyclonal, T cells bearing the same TCR and therefore allowing us to evaluate the impact of Ag dose on CD8^+^ T cell response while maintaining TCR affinity constant. In a polyclonal repertoire, an increase in Ag concentration may induce a stronger CD8^+^ T cell response by recruiting T cells with lower affinity for Ag, thereby yielding a different interpretation of the results. In our model, epitope density selectively affected CD8^+^ Tm cell generation. Possible explanations for this difference are increased inflammation and more sustained antigenic presentation during infection. Yet, it is possible that the signals required for CD8^+^ Tm cell generation are different when the level of inflammation is high. Furthermore, this is an agreement with the study of Joshi et al. [8] who have shown that the level of inflammation dictates the fate of effectors into short-lived cells or memory precursors. Indeed, effector CD8^+^ T cells obtained with our immunization protocol have a memory precursor phenotype (CD62L^hi^, IL-7R^hi^ and KLRG-1^lo^) and that they have also very good effector functions such as killing and cytokine production. Moreover, even though immunization with DCs expressing low or high level of the Ag leads both to the generation of CD8^+^ T cells with a memory precursor phenotype, it is intriguing that the efficiency of CD8^+^ Tm cell generation is different between the two immunized groups. Therefore, a different threshold of TCR engagement/signaling is required for the generation of short-lived effectors and long-lived CD8^+^ Tm cells.

More recently, Zehn et al. have shown using altered peptide ligands that very low affinity ligand are able to induce the full differentiation program of naïve CD8^+^ T cells leading to the generation of effector and memory T cells [6]. However, reduction of TCR ligand affinity impacted clonal burst-size [6]. Therefore, these experiments suggest that the strength of TCR signaling only influences the expansion phase of the T cell response without affecting CD8^+^ Tm cell development. Our results contrast with these data since we showed that a two-fold reduction in the amount of Ag presented to naïve CD8^+^ T cells, which also decreased TCR signal strength and TCR avidity, had a strong impact on CD8^+^ Tm cell differentiation. We would like to propose that naïve CD8^+^ T cells respond differently to a change in avidity resulting from a modification of TCR affinity for its ligand versus a change in avidity that occurs due to a variation of epitope density. Therefore, we would like to suggest that decreasing affinity will blunt T cell expansion while reducing epitope density will decrease CD8^+^ Tm cell generation. In a recent study, we have shown that lowering TCR expression level on naïve T cells influences T cell expansion and not memory generation [27]. However, to influence T cell expansion, the number of TCRs need to be reduced to a very low level (∼1000 molecules) [27]. With such a low level of TCR expression, it is more than likely that T cell expansion is reduced due to a decrease in T cell sensitivity that results from a loss of interaction with self-ligands known to be necessary to promote T cell responsiveness to foreign Ag [28], [29], [30], [31], [32], [33]. Thus, we would like to suggest that TCR density or affinity is involved in T cell expansion while epitope density influences memory generation.

Furthermore, it was shown by the group of Palmer that different TCR signals are required for the generation of effector versus memory CD8^+^ T cells [34] which is consistent with our results where a change in avidity, without a modification of affinity, only affected CD8^+^ Tm cell development. Thus, it is possible that a change in the avidity of the interaction will lead to a more optimal TCR signals allowing for the generation of CD8^+^ Tm cells. In support of that is our demonstration that reduction of the number of MHC-peptide complexes presented by DCs to naïve CD8^+^ T cells decreases both the duration of T-DC contact and the number of productive interaction as measured by the rate of T cell engulfment by DCs. Also, the level of the co-receptor CD8 engaged could also explain how a modification of avidity by manipulating Ag density affects memory generation while a change in avidity by varying affinity impinges on expansion. It is plausible that a change of TCR affinity will not influence the extent of CD8 engagement with MHC class I molecules while a variation of epitope density will.

The fact that our experimental system selectively affects the development of CD8^+^ Tm cells without influencing the number and function of effectors generated have allowed us to identify key molecular events required for CD8^+^ Tm cell generation. We have concentrated our effort on transcription factors known to control T cell differentiation such as T-bet, Eomes and Bcl6. Our results showed that the level of TCR engagement at priming selectively influences the expression of Eomes and Bcl6. This suggests that proper regulation of the expression of these two transcription factors is critical to induce CD8^+^ Tm cell differentiation. Although the variation of Eomes expression is only two-fold, we believe that this is enough to impact on CD8^+^ Tm cell differentiation since it was shown that the loss of one functional allele of Eomes is sufficient to reduce the size of the CD8^+^ Tm cell pool [17]. Moreover, small variations in the expression of a transcription factor will probably have a strong impact by affecting the transcription of multiple target genes. Furthermore, our results suggest that Eomes expression is not only controlled by inflammation level [18] and that the strength of TCR signaling also influences its expression. However, we cannot exclude that the longer duration of T-DC interaction might allow the DCs to provide a signal that will in turn up-regulate Eomes expression. As reported by Joshi et al. the level of TCR engagement by naïve CD8^+^ T cells did not influence the expression level of T-bet [8]. Furthermore, effectors unable to further differentiate into CD8^+^ Tm cells express slightly lower level of Bcl6 than the one that will generate memory. Since Bcl6 is essential for Tm cell development [20], [21], our results suggests that the strength of TCR signaling will influence Bcl6 expression and that the lack of memory generation with low dose of Ag could result from insufficient induction of Bcl6 expression. Since Bcl6 is not the mostly affected gene, our results suggest that induction of Bcl6 alone is not sufficient to promote CD8^+^ Tm cell differentiation without the cooperation of other transcription factors such as Eomes. In summary, the dose of Ag critically affects the generation of CD8^+^ Tm cells possibly by modulating the expression of Eomes and Bcl6 at the peak of the response. Although the number of CD8^+^ Tm cells was reduced more than seven-fold, the impact on transcription factor expression was less important suggesting that small variation in their expression is dramatic for the generation of CD8^+^ Tm cells. Such strong effects have also been reported in other biological systems where mice express only one functional allele of Eomes [17] and Bcl6 [35].

One effect of the strength of TCR signaling or of longer T-DC interaction on CD8^+^ Tm cell development is to control Nor-1 expression level to allow for a better survival of Ag-specific T cells during contraction. Reducing the density of Ag presented to naïve CD8^+^ T cells led to a higher level of Nor-1 expression. Furthermore, the 2-fold increase in Nor-1 expression correlates with a 1.6-fold increase in Bcl2-BH3 staining and is consistent with previous studies showing that Nor-1 can translocate to the mitochondria to convert Bcl-2 into a pro-apoptotic molecules [25].These results suggest that down-regulation of Nor-1 expression in effectors or memory precursors is essential to promote survival during differentiation into CD8^+^ Tm cells. Furthermore, our results indicate that caution should be taken when interpreting Bcl-2 expression level in activated T cells since Bcl-2 can also exist in an apoptotic form when Nor-1 is present. We would also like to propose that Nor-1 will play a similar role during CD4^+^ Tm differentiation. The group of Bevan has shown that Nor-1 and Bim are highly overexpressed in effectors in which memory differentiation is blunted [4], and we would like to suggest that Nor-1 will inhibit CD4^+^ Tm cell differentiation by inducing apoptosis of Ag-specific CD4^+^ T cells. Interestingly, we did not observed any change in Bim expression in our model. Our results suggest that the strength of TCR signaling does not influence survival of CD8^+^ effectors or memory precursors by regulating Bim expression but rather by controlling Nor-1 expression level. Although our results suggest that Nor-1 influences the survival of effectors by exposing the Bcl-2 BH3 loop, we cannot exclude that Nor-1 interferes with the survival/differentiation of effectors into CD8^+^ Tm cells at the transcriptional level since Nor-1 is also a transcription factor [23].

In summary, our data support a model where the epitope density sensed by the CD8^+^ T cell controls memory generation. Therefore, one important impact of our work relates to anti-tumor vaccination which relies on the use of DCs to immunize patients against tumor Ags. The dose of Ag presented by the DCs should be carefully analyzed to ensure proper differentiation of CD8^+^ Tm cells. Furthermore, analysis of the efficacy of the vaccination strategy should not only be performed at the effector stage of the response since the presence of effectors CD8^+^ T cells does not guarantee the proper generation of CD8^+^ Tm cells.

## Materials and Methods

### Mice

C57BL/6, B6.SJL, OT-1 and Vβ5LTAOCα^−/−^ mice were bred and maintained under specific pathogen-free condition. OT-1 were on a RAG-deficient background while Vβ5LTAOCα^−/−^ were bred to Cα-deficient mice. OT-1 and Vβ5LTAOCα^−/−^ express the same TCR specific for the OVA_257–264_ (SIINKEKL) peptide in the context of K^b^ [11], [12]. Mouse experiments were approved by the Animal Care Committee of the Maisonneuve-Rosemont Hospital Research Center (protocols 2009-02, 2009-21 and 2007-09) and mice were treated according to the guidelines of the Canadian Council on Animal Care.

### Antibodies (Abs) and cytometry

Anti-CD8, -KLRG1, -Ly6C, -CD122 and rat anti-mouse IgG1 Abs were purchased from BD Bioscience. Anti-perforin, -granzyme B, -CD127 Abs were from eBioscience. Anti-IL-2, -TNFα, -CD45.2, -4-1BB, -CD43, -CD5, -CD44, -CD62L and -CCR7 Abs were purchased from Biolegend Laboratories. Anti-IFNγ, -Sca-1, and -CD25 Abs were from Caltag Laboratories. Anti -Bcl-2-BH3 Ab was purchased from Abgent and anti-T-bet Ab from Santa Cruz Biotechnology. Fluorescently labeled streptavidins were purchased from BD Bioscience. Staining and analysis were done as described previously [9]. For Bcl-2 BH3 staining, cells were stained using the cytofix/cytoperm kit (BD Bioscience) [25].

### Preparation of peptide-pulsed DCs

DCs were generated as described previously [9]. To induce DC maturation, 1 µg/ml lipopolysaccharide (LPS) (Sigma-Aldrich) was added on day 6. The cells were either pulsed with OVA peptide (SIINFEKL; 2 µg/ml) at day 6 overnight (DC O/N) or for 3 h (DC 3 h) at day 7 to keep both DC subsets for the same amount of time in culture. The level of peptide-MHC complex was evaluated before immunization using the 25.D1.16 Ab specific for the K^b^-OVA complex [10]. As a control, the irrelevant peptide SIYRYYGL was used.

### Adoptive transfer, immunization and analysis of T cell response

10^6^ (unless otherwise stated) LN CD8^+^ T cells from female Vβ5LTAOCα^−/−^ or OT-I (CD45.2^+^) mice were injected i.v. into female B6.SJL (CD45.1^+^) hosts. 2 days later, mice were immunized i.v. with 5×10^5^ male B6.SJL mature peptide-pulsed DCs. At different days post- immunization, brachial or inguinal LNs were removed surgically and sequentially to follow response in the same mouse. Effector functions and response in tertiary sites were analyzed as described previously [9].

### Quantitative analysis of gene expression

Total RNA was isolated from sorted CD8^+^CD45.2^+^ cells at day 4 post-immunization and reverse transcribed into cDNA. Real-time PCRs were performed using SYBR Green (Invitrogen) on an Applied Biosystems 7500 real Time PCR system. The primer sequences used are listed in Table S1. The ΔC_T_ value (relative expression) for each sample was determined by calculating the difference between the C_T_ value of the target and the C_T_ value of the endogenous reference gene (HPRT).

### Time-lapse microscopy

Poly L Lysine-treated coverslips were treated 1 h with fibronectin type III (Sigma-Aldrich). 2×10^5^ DCs were then added and incubated in CO_2_ independent medium (Invitrogen) at 37°C into an imaging chamber. 2×10^5^ OT-1 T cells were added and sequential differential interference contrast images were recorded every 10 s over 1 h beginning one min after the addition of T cells on a Zeiss Z.1 inverted fluorescence microscope with CCD camera using the Northeclipse aplication from Empix Imaging.

### Statistical analysis

Statistics were done using Student's *t* or Mann-Whitney tests.

## Supporting Information

Figure S1 Phenotype of DCs loaded O/N or 3 h with the SIINFEKL peptide. (A) The bar chart shows the expression of CD86, MHC class I (Kb) and II (I-Ab) on LPS matured DCs loaded O/N (black bar) or 3 h (white bar) with SIINFEKL or on the surface immature DCs that were not loaded with antigen (dashed bar). (B) The bar chart shows the amount of IL-12 produced by LPS matured DCs loaded O/N (black bar) or 3 h (white bar) with SIINFEKL.
(0.19 MB TIF)

Figure S2 Generation of effector and memory CD8+ T cells after immunization with DCs expressing different amount of MHC-peptide complexes. DCs loaded O/N or for 3 h with the SIINFEKL peptide were used to immunize mice that have been adoptively transferred with OVA-specific naïve CD8+ T cells. T cell response was evaluated in the same mouse by surgical removal of LNs. As a negative control, mice were immunized with DCs loaded with an irrelevant peptide (DC IRR). The percentage and number of OVA-specific CD8+ T cells (CD45.2+) in one LN are indicated in each dot plot. One representative experiment out of 10 is shown.
(7.76 MB TIF)

Figure S3 Number of OVA-specific CD8+ T cells in tertiary sites. The bar charts show the number of OVA-specific T cells (CD8+CD45.2+) recovered in the different sites at d4 (A) or d75 (B) post-immunization with DCs loaded O/N (black bar) or 3 h (white bar) with the SIINFEKL peptide. (C) The relative number of OVA-specific CD8+ Tm cells recovered from the spleen at day 60 post-imunization with DC O/N (black bar) or DC 3 h (white bar) is shown.
(12.15 MB TIF)

Figure S4 Phenotype of memory and effector CD8+ T cells generated with different Ag doses. (A) Acquisition of a central memory phenotype by Ag-specific CD8+ T cells. Dot plots show CD62L/CD44 expression by OVA-specific CD8+ Tm cells 45d post-immunization with DCs expressing low (DCs 3 h) or high (DCs O/N) level of Ag while the overlay shows CD127 expression. (B–C) Similar phenotype of effectors. (B) The overlays show expression of activation markers, cytokine receptors, migration marker and memory marker by OVA-specific CD8 T cells 4 d post-immunization with DCs loaded with low (DCs 3 h) or high (DCs O/N) level of Ag. Representative of 6 independent experiments with 2–3 mice per group. (C) To normalize between experiments, the data are presented as a ratio of MFI of the CD8+CD45.2+ T cells (SIINFEKL-specific effectors) over the MFI of CD8+CD45.2- T cells (endogenous population). Each dot represents a mouse.
(0.40 MB TIF)

Figure S5 Epitope density does not affect the clonal burst size of OVA-specific CD8+ T cells when a low frequency of naïve T cell precursors is adoptively transferred. 104 CD8+ (CD45.2+) T cells from OT-1 mice were transferred into B6.SJL hosts (CD45.1+). Two days later, mice were immunized with 5 X 105 mature DCs loaded overnight (DC O/N) or 3 hours (DC 3 h) with the SIINFEKL peptide. On days 4, 6 and 7 after immunization, responsive CD8+ T cells were detected in blood, lymph node (LN) and spleen by staining with anti-CD45.2 and anti-CD8 Abs.
(3.86 MB TIF)

Figure S6 Effector functions are not affected by the amount of Ag seen at priming. (A) The percentage of specific lysis is shown for effectors obtained after immunization with DC pulsed with SIINFEKL O/N (DC O/N) or 3 h (DC 3 h). CFSE-labeled splenocytes pulsed or not with SIINFEKL were injected at d4 post-immunization. After 4 h, the presence of target cells was analyzed in the spleen. (B) The overlays show perforin (left) and granzyme B (right) staining for OVA-specific CD8+ T cells (CD8+CD45.2+, thick line) and recipient naïve T cells (CD8+CD45.2-, thin line) at d4 post-immunization. (C) The graph shows the MFI of perforin and granzyme B stainings at the peak of response. Each dot represents one mouse. (D) The histograms show IL-2 (left), TNF-alpha (middle) and IFN-gamma (right) production by OVA-specific CD8+ T cells (CD8+CD45.2+, thick line) and recipient T cells (CD8+CD45.2-, thin line) at d4 post-immunization. (E) Quantification of the amount of IL-2, TNF-alpha and IFN-gamma produced by the two groups of effectors. The MFI of cytokine production by effector CD8+ T cells was normalized to the MFI of the recipient CD8+ T cells. Results from panel A to E are from 2 independent experiments with 3 mice per group.
(0.37 MB TIF)

Figure S7 The level of antigenic presentation to naïve CD8+ T cells does not affect effector T cell survival. (A) Similar expression of Bcl-2 in effectors. The overlay represents Bcl-2 expression at the peak of response (d4) in CD8+CD45.2+ T cells obtained after immunization with DCs loaded O/N (DC O/N) or 3 h (DC 3 h). Dashed line, endogenous population (CD8+CD45.2-); dotted line, isotype control. (B) Kinetic of Bcl-2 expression. Bcl-2 expression is shown over time for effector CD8+ T cells generated after immunization with DCs loaded O/N (DC O/N) or 3 h (DC 3 h) with the OVA peptide. The MFI of Bcl-2 expression by effector CD8+ T cells was normalized to the MFI of the recipient CD8+ T cells. 2 independent experiments with 3 mice per group. (C) Expression of pro- and anti-apoptic molecules by effector CD8+ T cells. Effectors were sorted from spleen and LNs at d4 post-immunization to perform qPCR. The relative expression of the different genes after immunization with DC O/N or with DC 3 h is shown (normalized to HPRT). 3 independent experiments with 3 mice per group.
(4.31 MB TIF)

Table S1 (0.04 MB DOC)
