# Supplementary figures and images for: Epitope Density Influences CD8+ Memory T Cell Differentiation
Source: PLoS One. 2010 Oct 29;5(10):e13740. doi: 10.1371/journal.pone.0013740 (PMC2966420; doi:10.1371/journal.pone.0013740)

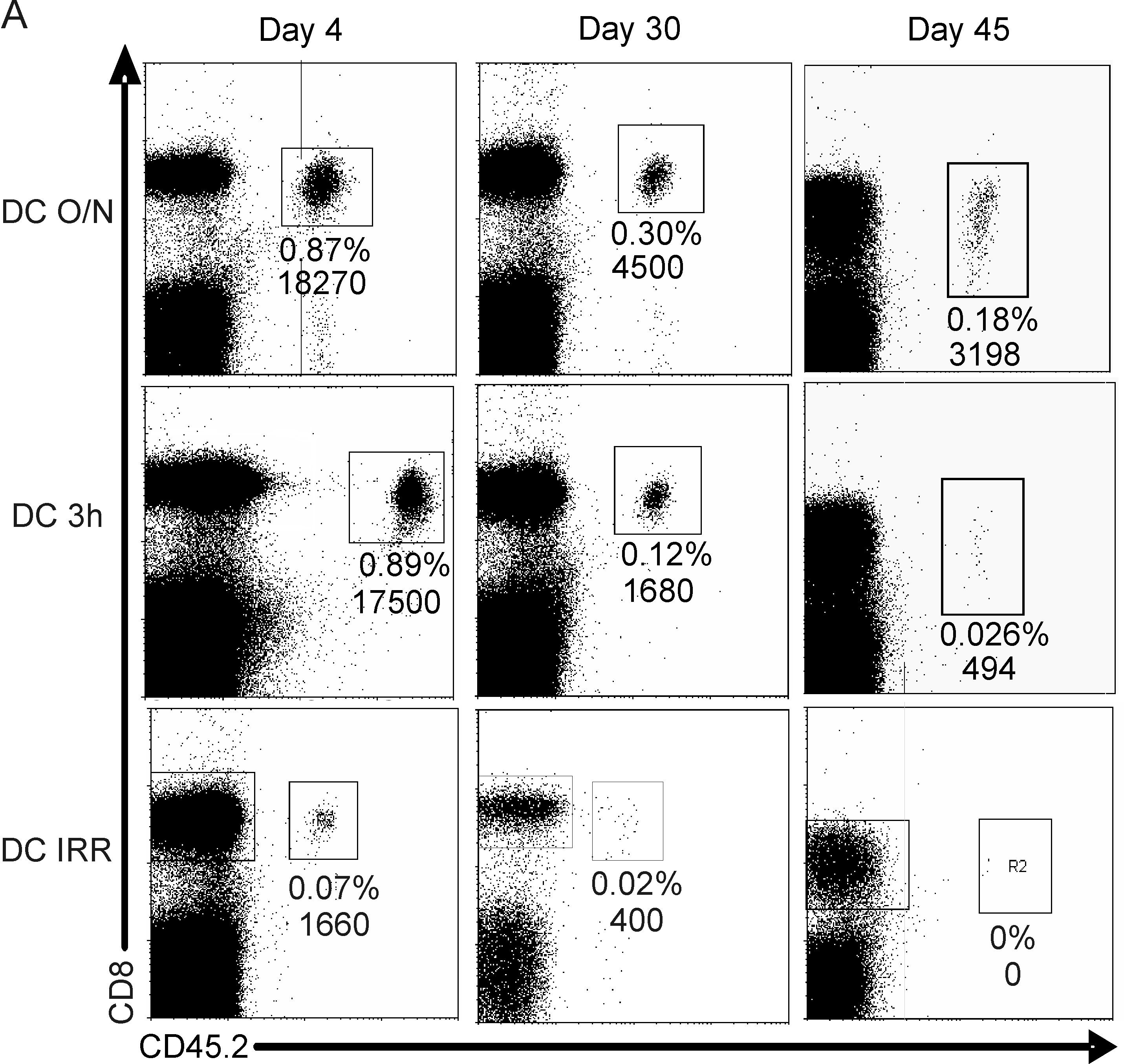

Supplement: Figure S2 — Generation of effector and memory CD8+ T cells after immunization with DCs expressing different amount of MHC-peptide complexes. DCs loaded O/N or for 3 h with the SIINFEKL peptide were used to immunize mice that have been adoptively transferred with OVA-specific naïve CD8+ T cells. T cell response was evaluated in the same mouse by surgical removal of LNs. As a negative control, mice were immunized with DCs loaded with an irrelevant peptide (DC IRR). The percentage and number of OVA-specific CD8+ T cells (CD45.2+) in one LN are indicated in each dot plot. One representative experiment out of 10 is shown. (7.76 MB TIF) [file pone.0013740.s002.tif]

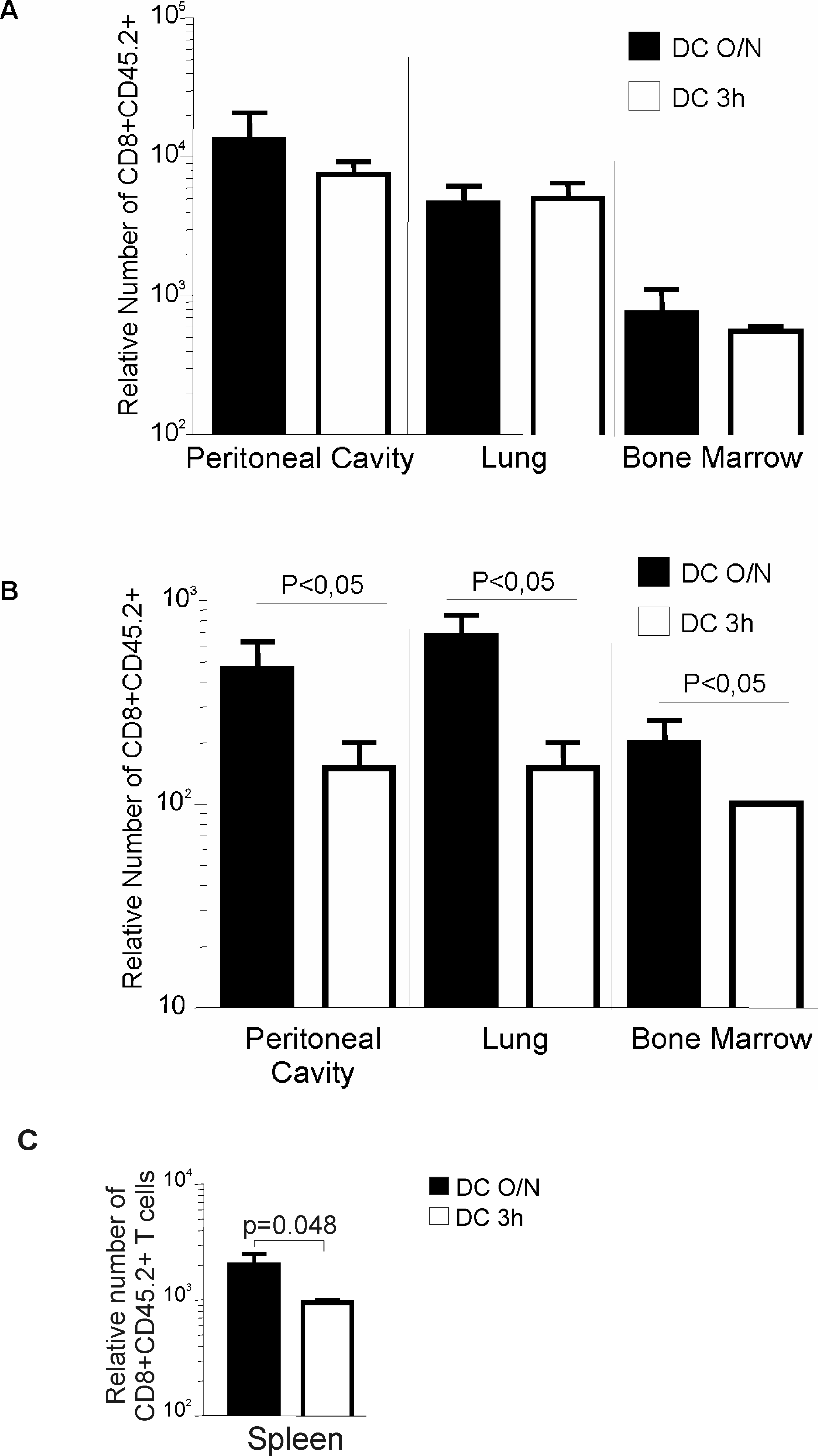

Supplement: Figure S3 — Number of OVA-specific CD8+ T cells in tertiary sites. The bar charts show the number of OVA-specific T cells (CD8+CD45.2+) recovered in the different sites at d4 (A) or d75 (B) post-immunization with DCs loaded O/N (black bar) or 3 h (white bar) with the SIINFEKL peptide. (C) The relative number of OVA-specific CD8+ Tm cells recovered from the spleen at day 60 post-imunization with DC O/N (black bar) or DC 3 h (white bar) is shown. (12.15 MB TIF) [file pone.0013740.s003.tif]

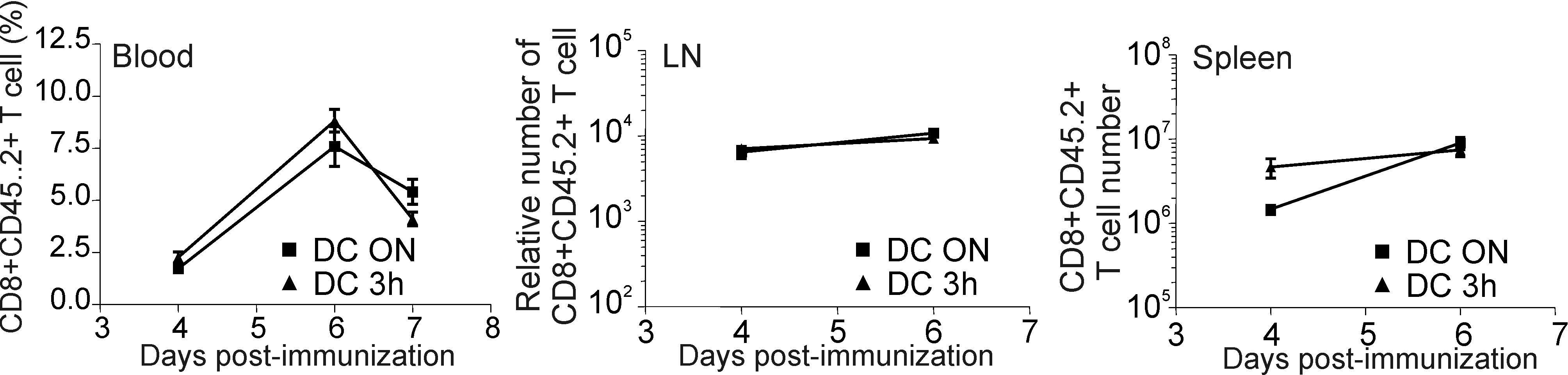

Supplement: Figure S5 — Epitope density does not affect the clonal burst size of OVA-specific CD8+ T cells when a low frequency of naïve T cell precursors is adoptively transferred. 104 CD8+ (CD45.2+) T cells from OT-1 mice were transferred into B6.SJL hosts (CD45.1+). Two days later, mice were immunized with 5 X 105 mature DCs loaded overnight (DC O/N) or 3 hours (DC 3 h) with the SIINFEKL peptide. On days 4, 6 and 7 after immunization, responsive CD8+ T cells were detected in blood, lymph node (LN) and spleen by staining with anti-CD45.2 and anti-CD8 Abs. (3.86 MB TIF) [file pone.0013740.s005.tif]

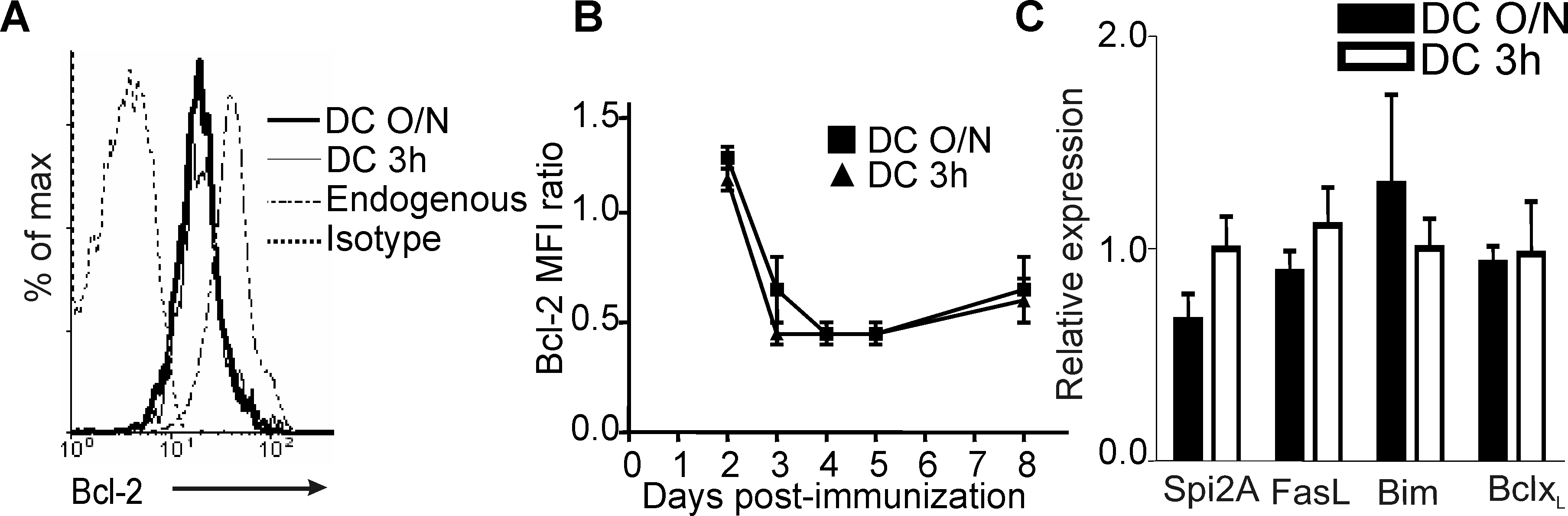

Supplement: Figure S7 — The level of antigenic presentation to naïve CD8+ T cells does not affect effector T cell survival. (A) Similar expression of Bcl-2 in effectors. The overlay represents Bcl-2 expression at the peak of response (d4) in CD8+CD45.2+ T cells obtained after immunization with DCs loaded O/N (DC O/N) or 3 h (DC 3 h). Dashed line, endogenous population (CD8+CD45.2-); dotted line, isotype control. (B) Kinetic of Bcl-2 expression. Bcl-2 expression is shown over time for effector CD8+ T cells generated after immunization with DCs loaded O/N (DC O/N) or 3 h (DC 3 h) with the OVA peptide. The MFI of Bcl-2 expression by effector CD8+ T cells was normalized to the MFI of the recipient CD8+ T cells. 2 independent experiments with 3 mice per group. (C) Expression of pro- and anti-apoptic molecules by effector CD8+ T cells. Effectors were sorted from spleen and LNs at d4 post-immunization to perform qPCR. The relative expression of the different genes after immunization with DC O/N or with DC 3 h is shown (normalized to HPRT). 3 independent experiments with 3 mice per group. (4.31 MB TIF) [file pone.0013740.s007.tif]
