# Supplementary material for: Epitope Density Influences CD8+ Memory T Cell Differentiation
Source: PLoS One. 2010 Oct 29;5(10):e13740. doi: 10.1371/journal.pone.0013740 (PMC2966420; doi:10.1371/journal.pone.0013740)
Supplement: Table S1 — (0.04 MB DOC) [file pone.0013740.s008.doc]

**Table S1. Real-time PCR primers**

| **Gene** | **Forward** | **Reverse** | **Reference** |
| --- | --- | --- | --- |
| Eomes | GTTTTCGTGGAAGTGGTTCTG | GGAGCCAGTGTTAGGAGATTC | 1 |
| Spi2A | AACCAGACCCTGAGGAAGTG | AACTTGGGCAGGCGGAG | 1 |
| Bcl6 | CCGGCTCAATAATCTCGTGAA | GGTGCATGTAGAGTGGTGAGTGA | 2 |
| T-bet | CCTGCAGTGCTTCTAACACACAC | CTCCGCTTCATAACTGTGTTC | 3 |
| Bim | CGGATCGGAGACGAGTTCA | TTCAGCCTCGCGGTAATCA | 4 |
| Nor-1 | GATCACAGAGCGACATGGGTTA | GAGCCTGTCCCTTCCTCTGG | 4 |
| FasL | TGAATTACCCATGTCCCCAG | AAACTGACCCTGGAGGAGCC | 5 |
| Bcl-x_L_ | GAGCTGGTGGTCGACTTTC | CTCCCTCTCTGCTTCAGTTTC | 1 |
| HPRT | CTCCTCAGACCGCTTTTTGC | TAACCTGGTTCATCATCGCTAATC | 3 |

1. Bachmann, M.F., R.R. Beerli, P. Agnellini, P. Wolint, K. Schwarz, and A. Oxenius. 2006. Long-lived memory CD8+ T cells are programmed by prolonged antigen exposure and low levels of cellular activation. *Eur. J. Immunol.* 36:842-854.
2. Yoshida, K., A. Sakamoto, K. Yamashita, E. Arguni, S. Horigome, M. Arima, M. Hatano, N. Seki, T. Ichikawa, and T. Tokuhisa. 2006. Bcl6 controls granzyme B expression in effector CD8+ T cells. *Eur. J. Immunol.* 36:3146-3156.
3. Intlekofer, A.M., N. Takemoto, E.J. Wherry, S.A. Longworth, J.T. Northrup, V.R. Palanivel, A.C. Mullen, C.R. Gasink, S.M. Kaech, J.D. Miller, L. Gapin, K. Ryan, A.P. Russ, T. Lindsten, J.S. Orange, A.W. Goldrath, R. Ahmed, and S.L. Reiner. 2005. Effector and memory CD8+ T cell fate coupled by T-bet and Eomesodermin. *Nat. Immunol.* 6:1236-1244.
4. Williams, M.A., E.V. Ravkov, and M.J. Bevan. 2008. Rapid culling of the CD4+ T cell repertoire in the transition from effector to memory. *Immunity* 28:533-545.
5. Pinkoski, M.J., N.M. Droin, T. Lin, L. Genestier, T.A. Ferguson, and D.R. Green. 2002. Nonlymphoid Fas ligand in peptide-induced peripheral lymphocyte deletion. *Proc. Nat. Acad. Sci. USA* 99:16174-16179.
